# Factors associated with sugar-sweetened beverage consumption in adults with children in the home after a statewide health communications program

**DOI:** 10.1186/s40795-020-00349-4

**Published:** 2020-06-17

**Authors:** Shirley A. James, Ashley H. White, Sjonna Whitsitt Paulson, Laura A. Beebe

**Affiliations:** 1 Department of Biostatistics and Epidemiology, Hudson College of Public Health, University of Oklahoma Health Sciences Center, 801 NE 13th Street, Oklahoma City, OK 73104 USA; 2 Oklahoma Tobacco Settlement Endowment Trust, 2800 N. Lincoln Blvd, Suite 202, Oklahoma City, OK 73105 USA

**Keywords:** Sugar-sweetened beverages, Obesity, Healthy diets, Media campaign, Mass media, Soda consumption, Knowledge, Attitudes

## Abstract

**Background:**

In 2016, Oklahoma launched “Shape Your Future – Rethink Your Drink” (SYF/RYD), an obesity prevention health communication program targeting parents and caregivers of children. The aims of this study are to compare sugar-sweetened beverage (SSB) consumption before and after the program and to report factors associated with SSB consumption, knowledge, and attitudes.

**Methods:**

This repeated cross-sectional study involved 2656 Oklahoma adults with ≥ one child under 18 years in the home. Weighted prevalence estimates were calculated and the relationship between SSB consumption and covariates of interest were examined using logistic regression techniques appropriate for survey data.

**Results:**

Following the SYF/RYD program, SSB consumption decreased 18.6% (*p* = 0.0232) and heavy SSB consumption, ≥ three SSB per day, decreased 42.9% (*p* = 0.0083). Factors associated with SSB consumption, 1 year after the launch of SYF/RYD included high school education or less (AOR = 1.33 with 95% CI = 1.02, 1.73), fair or poor health status (AOR = 2.02 with 95% CI = 1.47, 2.78), drinking less than eight cups of water daily (AOR = 1.77 with 95% CI = 1.39, 2.25), inability to afford healthy foods (AOR = 1.33 with 95% CI = 1.06, 1.67), and self-identifying as American Indian/Alaska Native (AOR = 1.59 with 95% CI = 1.10, 2.29).

**Conclusions:**

Health communication campaigns, such as SYF/RYD, are an evidence-based strategy for health behavior change and likely contributed to the declines observed in SSB consumption. Important differences in SSB consumption by population subgroups persist and have implications for future message development.

## Background

The 2018 Behavioral Risk Factor Surveillance System (BRFSS) data reported Oklahoma’s prevalence of obesity among adults at 35% [1]. Additionally, nearly 19% of children 10 to 17 years of age are obese, making Oklahoma the fifth highest state in terms of childhood obesity [2]. Important obesogenic factors include culture, home environment, corporate advertising, and parental knowledge [3]. Because parents can only initiate healthy habits after they understand what those habits entail, parental knowledge has a strong influence on the diets of youth and children residing in the home, on their exercise habits, and on their beverage choices [3, 4]. Parental beverage choices, as well as beverage availability in the home, influence the quality of children’s diets [4–6]. Effects of these choices can continue from childhood to adulthood [7]. Furthermore, children whose parents or other caregivers encourage healthy eating and who model a healthy lifestyle are more likely to adopt healthy living patterns themselves [6, 7].

Research suggests that the consumption of sugar-sweetened beverages (SSBs) is associated with obesity in both adults and children [8]. SSBs include “any liquids sweetened with various forms of added sugars including brown sugar, corn sweetener, corn syrup, dextrose, fructose, glucose, high-fructose corn syrup, honey, lactose, malt syrup, maltose, molasses, raw sugar, and sucrose.” [9] Examples of SSBs include regular soda (not sugar-free), fruit drinks, sports drinks, energy drinks, sweetened waters, and coffee and tea beverages with added sugars. Intake of SSBs is positively associated with both increased body weight and risk of obesity, and is negatively associated with the intake of important micronutrients [10].

Social marketing and public health mass media campaigns have been effective in changing health behaviors, knowledge, and attitudes [11–16], and are considered an evidence-based strategy [17]. Specifically, media campaigns targeting the consumption of SSBs have had success influencing individual *intentions* to change behavior [11, 12], and initiate changes in SSB consumption in children and adults [17]. Further, these campaigns have influenced media coverage and reporting of SSB consumption [18]. To date, few research studies have examined changes in SSB consumption after media campaigns in the population of adults with children in the home. Additionally, the need to examine SSB consumption in combination with attitudes and knowledge about SSB consumption is critical to inform and contribute to the success of future SSB consumption reduction campaigns.

In 2016, the Oklahoma Tobacco Settlement Endowment Trust (TSET) launched an ongoing obesity prevention health communication program aimed at Oklahoma parents and caregivers of children, ‘Shape Your Future: Rethink Your Drink (SYF/RYD),‘a program which continued through 2017 and beyonod [19]. The program developed and released messages designed to combat SSB consumption by urging Oklahomans to ‘Rethink Your Drink,’ and replace SSBs with water. Additionally, the program was designed to educate Oklahomans about the health effects associated with SSB consumption and reasons to limit SSB consumption for their families. The “Rethink Your Drink” program and messages originated from the Nutrition Education and Obesity Prevention Branch of the California Department of Public Health [12, 20].

We previously reported the results of a 2015 cross-sectional study designed to gather information about Oklahomans’ knowledge, attitudes, and behaviors concerning SSBs [19]. The study reported baseline SSB consumption in Oklahoma adults with children living in the home prior to the launch of the ‘Shape Your Future - Rethink Your Drink’ (SYF/RYD) health communication program. The current study reports the results of a second cross-sectional study, initiated after the first quarter of the campaign. The objectives of this analysis were four-fold; first, to report post-campaign SSB consumption by type of beverage and overall; second, to compare SSB consumption before and after the SYF/RYD program launch; third, to measure exposure to the SYF/RYD program; and fourth, to measure covariates impacting SSB consumption by type of beverage and overall. The purpose of this study was to provide data about adult consumption of SSBs as well as knowledge and attitudes toward SSB consumption after the launch of the statewide media program, ‘Shape Your Future: Rethink Your Drink’ (SYF/RYD).

## Methods

Methodology from the 2015 survey, which occurred before the launch of SYF/RYD, has been reported in another paper [19], and survey questions were identical for both time periods, using previously validated survey items for SSB consumption [14–16]. TheSYF/RYD campaign was launched in July 2016, with combined television and cable outlets, digital print, radio, bulletins, and posters. The program is ongoing. Data collection after the program launch occurred October 2016 through July 2017 via telephone survey by the Sooner Survey Center at the University of Oklahoma Hudson College of Public Health. The population was a random sample of all non-institutionalized adults in Oklahoma with at least one child under the age of 18 years living in the household and with either a cellular or landline telephone. Data were weighted to adjust for non-coverage and non-response, creating estimates more representative of the Oklahoma adult population with children living in the home. The survey instrument and protocol were approved by the University of Oklahoma Health Sciences Center Institutional Review Board.

### SSB outcome variables

We measured SSB intake by first asking respondents, “*During the past 30 days, how often did you drink regular soda or pop that contains sugar?”* We then asked, *“During the past 30 days, how often did you drink sugar-sweetened fruit drinks, sweet tea, sports drinks, or energy drinks?”* Respondents were asked to NOT include 100% fruit juice, diet drinks, or artificially sweetened drinks. For both questions, respondents could answer in times per day, week, or month. We converted responses to average daily intake, and examined daily SSB consumption for soda and other SSBs separately and in combination. This analysis includes four outcomes related to SSB consumption. First, consumption of any SSB; second, consumption of sugar sweetened sodas (SS sodas); and third, consumption of other SSBs (not including soda), each defined as one or more beverages per day. The fourth outcome was heavy SSB consumption, which we defined as consumption of three or more SSBs of any type per day [19, 21].

### SSB knowledge and attitude variables

This study included three questions designed to measure knowledge and attitudes toward SSB consumption. The first two attitude questions were intended to measure behavioral intent to change with answers to these statements: *I am able to substitute water for SSBs for my family* and *I plan to limit SSBs for my family*. The third question was intended to measure knowledge about the consequences of SSB consumption with answers to this statement: *SSBs are linked to obesity, diabetes, and heart disease.* We categorized answers to all three items into two levels, ‘strongly agree’ versus a combined category including somewhat agree, disagree, and strongly disagree.

### SYF/RYD campaign exposure

We measured confirmed exposure to the SYF/RYD program by first asking respondents, “Have you seen or heard any ads in the past month about replacing sugar sweetened drinks with water?” If yes, they were asked what the theme, name, or slogan of this program was, and to describe an ad. Respondents were considered to have confirmed exposure if they named the slogan as SYF or RYD or described a SYF/RYD advertisement.

### Covariates

Covariates included gender, three categories of age (< 35 years, 35–54 years, and ≥ 55 years), four categories of race (White, American Indian/Alaska Native, African American and “other”), two levels of education (high school degree or less versus some college /technical school or more), and two self-assessed levels of general health (excellent, very good, or good, versus fair or poor). Other covariates included daily water consumption (eight or more cups versus 0–7 cups), daily fruit servings (three or more versus less than three), daily vegetable servings (three or more versus less than three), two categories of moderate to intense physical exercise (three or more days per week versus less than three) and availability of healthy food, “I am able to afford healthy foods” (strongly agree versus somewhat agree, disagree, or strongly disagree combined).

### Data analysis

Post-stratification weights were calculated along dimensions of age, gender, race, income, and education using American Community Survey data to adjust for non-response and sources of under-coverage. Imputation was used for missing responses in raking dimension variables [22, 23]. We used SAS version 9.4, for all analyses. Survey procedures and sampling weights were used to obtain population-level estimates unless indicated otherwise. Pearson chi-square tests were used to examine whether the outcomes varied by the covariates of interest. *P*-values < 0.05 were considered statistically significant. Separate multivariable logistic regression analyses were performed for the SSB outcomes, modeling relationships between outcome variables and the covariates described above. Variables in the model were removed using a manual stepwise method, with decisions based on the t-statistics for estimated coefficients, using the associated *p*-values. Adjusted odds ratios with confidence intervals are reported. Logistic regression equations used unweighted data in an attempt to provide unbiased parameter estimates with smaller standard errors, more accurately representing the variables that impact SSB consumption in this particular sample [24, 25].

## Results

The target population for the SYF/RYD program was Oklahoma adults with children living in the home. In 2015, prior to the launch of the SYF/RYD media program, we surveyed 1118 Oklahomans. After the launch of the SYF/RYD program, we surveyed 1538 Oklahomans. The American Association for Public Opinion Research (AAPOR) response rate (RR1) was 10% in 2015 and 5% in 2017; the cooperation rates were 90 and 89% respectively, both within acceptable ranges for both survey periods [26, 27]. The demographic characteristics during both time periods were similar and included predominantly female (55% in 2015 versus 54% in 2017) and white (66% versus 68%), with about half (47%) reporting having a high school education or less. About two-thirds (69% versus 63%) reported they participated in moderate to intense physical exercise three or more days every week. While most (82% versus 85%) perceived their health status as excellent, very good, or good, only half or less (50% versus 42%) drank the recommended eight cups of water or more daily, and less than one quarter ate the recommended daily three or more servings of fruits (23% versus 20%) and vegetables (19% versus 21%) [28]. Only one third strongly agreed they could afford to buy healthy foods in 2015 (34%), compared to about half (48%) in 2017. Statistically significant differences between 2015 and 2017 survey results existed for only two variables, daily water consumption (*p* < 0.05) and ability to afford healthy food (*p* < 0.0001) (Table 1).

**Table 1:** Demographic, health characteristics, and confirmed exposure to the Shape Your Future/Rethink Your Drink Campaign

**Characteristic**	**n (weighted %) 2015**	**n (weighted %) 2017**	**Confirmed Exposure (weighted % and 95 CI) 2017**
Overall	1118	1538	23.8% (20.0, 27.6)
Gender			
Male	348 (45.3)	630 (46.4)	17.6 (13.2, 22.0)^a^
Female	767 (54.7)	904 (53.6)	29.1 (23.3, 34.8)^a^
missing	3	4	
Age (years)			
18-34-2015	180 (44.4)	593 (43.5)	24.4 (17.6, 31.2)
35-54-2015	783 (47.3)	785 (47.1)	23.9 (19.2, 28.5)
≥ 55–2015	155 (8.3)	159 (9.4)	20.8 (11.8, 29.8)
missing	0	1	
Race/Ethnicity			
White	883 (65.9)	1022 (67.7)	22.9 (19.2, 26.6)
Black	66 (8.3)	160 (7.9)	28.0 (17.9, 38.0)
American Indian	69 (9.0)	163 (7.3)	14.3 (8.3, 20. 3)
Other	89 (16.7)	187 (17.1)	29.3 (14.0, 44.6)
missing	11	6	
Education			
≤ High school	184 (47.3)	355 (47.1)	21.6 (16.1, 27.0)
> High school	915 (52.7)	1182 (52.9)	25.6 (20.4, 30.9)
missing	19	1	
Perceived health status			
Excellent, very good or good	973 (82.4)	1308 (84.6)	23.0 (19.3, 26.8)
Fair or poor	144 (17.6)	227 (15.4)	28.1 (15.2, 40.9)
missing	1	3	
Daily water consumption			
≥ 8 cups	509 (49.8)	573 (42.0)	25.9 (19.7, 32.1)
0–7 cups	608 (50.2)	963 (58.0)	22.3 (17.5, 27.0)
missing	1	2	
Daily servings of fruit			
≥ 3 servings	268 (22.6)	263 (20.1)	22.4 (14.2, 30.7)
< 3 servings	846 (77.4)	1249 (79.9)	24.7 (20.4, 29.1)
missing	4	26	
Daily servings of vegetables			
≥ 3 servings	238 (19.2)	321 (20.8)	18.6 (12.9, 24.2)
< 3 servings	877 (80.8)	1196 (79.2)	25.6 (21.0, 30.2)
missing	3	21	
Moderate to intense exercise			
≥ 3 days per week	740 (68.5)	973 (62.8)	24.2 (19.5, 28.9)
< 3 days per week	378 (31.5)	551 (37.2)	24.0 (17.4, 30.6)
missing	0	14	
Able to afford healthy food			
Strongly agree	427 (33.8)^b^	623 (47.7) ^b^	25.9 (19.6, 32.2)
Do not strongly agree	691 (66.2) ^b^	914 (52.3) ^b^	21.9 (17.5, 26.3)
missing	0	1	

^a^ indicates statistically significant exposure differences between men and women

^b^ indicates a statistically significant (*p* < 0.0001) difference in ability to afford healthy food between 2015 and 2017

### Consumption of SSBs

Following the SYF/RYD media program, the prevalence of daily consumption of any SSB was 36.3% compared to 44.6% in 2015 (*p* = 0.0232), a decrease of 19%. The prevalence of daily sugary soda consumption was 21.1% in 2017 compared to 29.4% in 2015 (*p* = 0.0119), a decrease of 28%. The prevalence of daily non-soda SSB consumption was 22.9% in 2017 compared to 29.3% in 2015 (*p* = 0.0543), a decrease of 22%, and the prevalence of heavy SSB consumption was 9.2% compared to 16.1% in 2015 (*p* = 0.0083), a decrease of 43% (Table 2).

**Table 2:** Sugar sweetened beverage (SSB) consumption, pre and post Shape Your Future/Rethink Your Drink (SYF/RYD) and by sociodemographic and health-related characteristics

**Characteristic**	**Pre SYF/RYD** **2015**	**Post SYF/RYD** **2017**	**Relative percent change** **from 2015 to 2017**
**Daily Consumption of one or more sugar sweetened sodas – (weighted percentages)**			
Overall	29.4% (23.5, 35.4)	21.1% (17.7, 24.5)	↓28.2% (*p* = 0.0119) ^a^
**Daily Consumption of one or more SSB other than soda - (weighted percentages)**			
Overall	29.3% (23.4, 35.2)	22.9% (19.3, 26.4)	↓21.8% (*p* = 0.0543)
**Heavy SSB Consumption (three or more SSBs per day) - (weighted percentages)**			
Overall	16.1% (10.8, 21.3)	9.2% (6.8, 11.6)	↓42.9% (*p* = 0.0083) ^a^
**Daily Consumption of one or more SSB of any kind – Weighted percentage with 95% CI**			
Overall	44.6 (38.6, 50.5)	36.3 (32.1, 40.5)	↓ 18.6% (*p* = 0.0232) ^a^
Gender			
Male	45.7 (36.6, 54.8)	37.5 (31.3, 43.7)	↓ 17.9% (*p* = 0.1333)
Female	43.1 (35.3, 50.9)	35.2 (29.4, 41.0)	↓ 18.3% (*p* = 0.1082)
Age (years)			
18-34-2015	49.1 (38.8, 59.3)	39.2 (31.9, 46.5)	↓ 20.2% (*p* = 0.1157)
35-54-2015	40.9 (33.6, 48.1)	33.0 (27.5, 38.5)	↓ 19.3% (*p* = 0.0866)
≥ 55–2015	41.3 (26.4, 56.1)	39.3 (27.7, 50.8)	↓ 4.8% (*p* = 0.8335)
Race/Ethnicity			
White	44.7 (37.6, 51.8)	37.9 (33.2, 42.6)	↓ 15.2% (*p* = 0.1100)
Black	31.4 (13.3, 49.4)	37.0 (25.6, 48.3)	↑ 17.8% (*p* = 0.6096)
American Indian	51.3 (33.4, 69.2)	42.1 (28.3, 55.9)	↓ 17.9% (*p* = 0.4252)
Other	47.0 (30.6, 63.3)	27.5 (14.2, 40.7)	↓ 41.5% (*p* = 0.0584)
Education			
> High school	30.3 (24.9, 35.6)	31.2 (26.2, 36.2)	↑ 3.0% (*p* = 0.8088)
≤ High school	**61.1 (51.4, 70.8)**	**42.0 (35.1, 48.8)**	↓ 31.3% (*p* = 0.0017)^a^
Perceived health status			
Excellent, very good or good	43.2 (36.5, 49.8)	**32.9 (28.6, 37.1)**	↓ 23.8% (*p* = 0.0078) ^a^
Fair or poor	49.5 (35.8, 63.2)	56.8 (45.1, 68.5)	↑ 14.7% (*p* = 0.4206)
Daily water consumption			
≥ 8 cups	**38.0 (29.1, 46.8)**	**26.1 (20.1, 32.0)**	↓ 31.3% (*p* = 0.0221) ^a^
0–7 cups	51.1 (43.4, 58.9)	43.8 (38.3, 49.3)	↓ 14.3% (*p* = 0.1307)
Daily servings of fruit			
≥ 3 servings	36.1 (22.6, 49.6)	28.6 (19.0, 38.2)	↓ 20.8% (*p* = 0.3622)
< 3 servings	47.1 (40.6, 53.7)	38.3 (33.6, 43.0)	↓ 18.7% (*p* = 0.0298) ^a^
Daily servings of vegetables			
≥ 3 servings	40.1 (27.6, 52.5)	31.4 (23.0, 39.8)	↓ 21.7% (*p* = 0.2477)
< 3 servings	45.8 (39.1, 52.5)	37.5 (32.6, 42.4)	↓ 18.1% (*p* = 0.0461) ^a^
Moderate to intense exercise			
≥ 3 days per week	43.0 (35.5, 50.4)	36.1 (31.1, 41.2)	↓ 16.0% (*p* = 0.1270)
< 3 days per week	48.0 (38.4, 57.5)	36.6 (29.0, 44.1)	↓ 23.7% (*p* = 0.0635)
Able to afford healthy food			
Strongly agree	**35.5 (26.1, 44.8)**	33.7 (27.62, 39.7)	↓ 5.1% (*p* = 0.6397.)
Do not strongly agree	49.2 (41.9, 56.5)	39.4 (33.6, 45.2)	↓ 19.9% (*p* = 0.0335) ^a^

Boldface indicates *within year* statistical significance (*p* < 0.05)

^a^ indicates significant change from 2015 to 2017

While there were no statistically significant differences in SSB consumption between 2015 and 2017 within subgroups by gender, age, or race, several approached significance (*p* < 0.10), Table 2). Conversely, among those with a high school education or less, the prevalence of daily SSB consumption decreased 31% from 61% in 2015 to 42% in 2017 (*p* < 0.01). Additionally, in those who perceived their health status as excellent, very good, or good, there was a statistically significant 24% decrease in SSB consumption between 2015 (33%) and 2017 (24%). Additional significant decreases in SSB consumption occurred in those who drink eight or more cups of water per day (31% ↓), who eat less than the recommended 3 servings of fruit (19% ↓) and vegetables (18% ↓) daily, and in those who are not able to afford healthy foods (20% ↓) (Table 2).

### SYF/RYD campaign exposure

About 24% of Oklahomans with children in the home demonstrated confirmed exposure to SYF/RYD (Table 1). While women were more likely to have confirmed exposure than men (29% versus 18%), the program reached similar proportions with respect to age, education, self-perceived health status, water consumption, dietary consumption of fruits and vegetables, weekly amount of physical exercise, and the ability to afford healthy food, a measure of economic status. One notable exception was race. Although there was not a statistically significant difference within that variable, confirmed exposure among American Indian/Alaska Natives was lower than all other groups (Table 1).

With respect to outcome measures, there were no significant differences in total SSB consumption, sugary soda consumption, or other SSB consumption by campaign exposure status. Similarly, there was no difference among those with and without confirmed campaign exposure in plans to limit SSB consumption for their families or perceived ability to substitute water for SSBs for their families. There was, however, a statistically significant difference in knowledge about the consequences of SSB consumption by exposure to the campaign. Among those with confirmed exposure, three quarters (76%) knew SSB consumption is linked to obesity, diabetes, and heart disease compared to 64% without confirmed exposure (*p* = 0.0045, Table 3).

**Table 3:** Sugar-sweetened beverage consumption, attitudes and knowledge by exposure to Shape Your Future/Rethink Your Drink, 2017

**RYD campaign-related outcomes**	**Confirmed Exposure to SYF/RYD**		***p*-value**
	**Yes** *N* = 383 23.8% (20.0, 27.6)	**No** *N* = 1155 76.2% (72.4, 80.0)	
One or more SSBs of any kind per day	36.7% (27.4, 45.9)	36.2 (31.4, 40.9)	0.9245
One or more SS sodas per day	19.5 (12.8, 26.1)	21.6 (17.6, 25.6)	0.5901
One or more SSBs other than soda/day	20.9 (14.0, 27.8)	23.5 (19.4, 27.6)	0.5418
Heavy SSB consumption (three or more SSBs of any kind/ day)	7.4 (3.4, 11.3)	9.8 (6.9, 12.7)	0.3564
I am able to substitute water for sugar-sweetened beverages for my family	64.0 (55.1, 72.8)	56.9 (52.1, 61.7)	0.1820
I plan to limit sugar-sweetened drinks for my family	55.2 (46.1, 64.2)	49.7 (44.7, 54.8)	0.3070
SSBs are linked to obesity, diabetes & heart disease	76.2 (69.8, 82.7)	64.1 (59.5, 68.8)	**0.0045**

Bold type indicates statistical significance at alpha = 0.05 level

### Factors associated with daily SSB consumption

The results of the multivariable logistic regression analysis of the 2017 survey data revealed a number of factors were independently associated with consuming one or more SSB of any kind per day. These included high school education or less (aOR = 1.33, 95% CI = 1.02, 1.73), perceived health status as fair or poor (aOR = 2.02, 95% CI = 1.47, 2.78), and inability to afford healthy foods (aOR = 1.33, 95% CI = 1.06, 1.67, Table 4). Other dietary behaviors were also associated with total SSB consumption including drinking less than eight cups of water per day (aOR = 1.77 with a 95% CI = 1.39, 2.25), fewer than three daily servings of fruits (aOR = 1.70 with a 95% CI = 1.23, 2.34) and fewer than three daily servings of vegetables (aOR = 1.35 with a 95% CI = 1.01, 1.81). After adjusting for other variables in the model, the odds of SSB consumption were also higher in American Indian/Alaska Natives compared to Whites (aOR = 1.59, 95% CI = 1.10, 2.29).

**Table 4:** Factors associated with SSB consumption in 2017 (adjusted OR and 95% CI)

	**Daily Consumption of One or more SSBs of Any Kind**	**Daily Consumption of One or more SS Sodas**	**Daily Consumption of One or More Other SSBs**	**Heavy SSB Consumption (Daily Consumption of three or more SSBs)**
Gender				
Male	1.19 (0.95, 1.50)	0.94 (0.72, 1.22)	**1.61 (1.24, 2.08)**	1.20 (0.83, 1.72)
Female	(ref)	(ref)	(ref)	(ref)
Age (years)				
18–34	1.48 (0.99, 2.21)	1.34 (0.86, 2.10)	1.18 (0.76, 1.84)	1.83 (0.93, 3.60)
35–54	1.04 (0.71, 1.53)	1.24 (0.80, 1.91)	0.83 (0.53, 1.28)	1.39 (0.71, 2.72)
≥ 55	(ref)	(ref)	(ref)	(ref)
Race/Ethnicity				
White	(ref)	(ref)	(ref)	(ref)
Black	1.01 (0.70, 1.45)	1.09 (0.73, 1.65)	1.14 (0.76, 1.70)	1.07 (0.58, 1.95)
American Indian	**1.59 (1.10, 2.29)**	**1.66 (1.12, 2.46)**	**1.57 (1.05, 2.34)**	**2.06 (1.27, 3.36)**
Other	0.81 (0.57, 1.15)	0.84 (0.56, 1.27)	0.77 (0.50, 1.18)	0.77 (0.42, 1.39)
Education				
> High school	(ref)	(ref)	(ref)	(ref)
≤ High school	**1.33 (1.02, 1.73)**	1.27 (0.94, 1.70)	**1.55 (1.16, 2.07)**	1.34 (0.90, 1.99)
Perceived health status				
Excellent, very good or good	(ref)	(ref)	(ref)	(ref)
Fair or poor	**2.02 (1.47, 2.78)**	1.33 (0.94, 1.89)	**1.82 (1.29, 2.58)**	**2.19 (1.42, 3.38)**
Daily water consumption				
≥ 8 cups	(ref)	(ref)	(ref)	(ref)
0–7 cups	**1.77 (1.39, 2.25)**	**1.79 (1.36, 2.36)**	**1.79 (1.37, 2.36)**	**2.12 (1.41, 3.18)**
Daily servings of fruit				
≥ 3 servings	(ref)	(ref)	(ref)	(ref)
< 3 servings	**1.70 (1.23, 2.34)**	**1.49 (1.02, 2.17)**	1.39 (0.96, 2.00)	1.12 (0.67, 1.86)
Daily servings of vegetables				
≥ 3 servings	(ref)	(ref)	(ref)	(ref)
< 3 servings	**1.35 (1.01, 1.81)**	1.31 (0.93, 1.85)	0.98 (0.71, 1.35)	1.14 (0.71, 1.83)
Moderate to intense exercise				
≥ 3 days per week	(ref)	(ref)	(ref)	(ref)
< 3 days per week	0.87 (0.68, 1.10)	1.03 (0.79, 1.34)	0.89 (0.67, 1.17)	1.30 (0.89, 1.88)
Able to afford healthy food				
Strongly agree	(ref)	(ref)	(ref)	(ref)
Do not strongly agree	**1.33 (1.06, 1.67)**	1.22 (0.94, 1.59)	1.30 (1.00, 1.70)	0.93 (0.65, 1.34)
Confirmed Exposure				
Yes	1.04 (0.81, 1.35)	0.97 (0.72, 1.30)	1.00 (0.75, 1.35)	0.88 (0.58, 1.35)
No	(ref)	(ref)	(ref)	(ref)

All ORs are adjusted for exposure, gender, age, race, education, health status, daily water fruit and vegetable consumption, daily exercise levels, and ability to afford healthy foods

Bold type indicates odds ratios without a one in the confidence interval

### Factors associated with daily soda consumption

When sugary soda was considered, three factors remained independently associated with daily consumption. The odds of sugar-sweetened soda consumption were higher for American Indian/Alaska Natives compared to Whites (aOR = 1.66, 95% CI = 1.12, 2.46) and in those who consumed less than the recommended eight cups of water daily (aOR = 1.79, 95% CI = 1.36, 2.36). Additionally, the odds of sugar-sweetened soda consumption were higher for those who did not meet the daily dietary recommendation of three servings of fruits (aOR = 1.49, 95% CI = 1.02, 2.17, Table 4).

### Factors associated with daily SSB consumption not including soda

Five factors were associated with increased odds of SSB consumption, other than soda. The odds of SSB consumption not including sodas were higher in men (aOR = 1.61, 95% CI = 1.24, 2.08), American Indian/Alaska Natives (aOR = 1.57, 95% CI = 1.05, 2.34), and among those with a high school education or less (aOR = 1.55, 95% CI = 1.16, 2.07). Additionally, fair or poor perceived health status (aOR = 1.82, 95% CI = 1.29, 2.58), and drinking fewer than eight cups of water a day (aOR = 1.79, 95% CI = 1.37, 2.36, Table 4) were significantly associated with SSB consumption not including sodas.

### Factors associated with heavy SSB consumption

Heavy consumption of any SSB was defined as three or more SSBs of any kind per day and was reported by 9.2% of Oklahomans with children in the home in 2017. After adjusting for other variables in the model, the odds of consuming three or more SSBs per day were twice as high among American Indian/Alaska Natives compared to White (aOR = 2.06, 95% CI = 1.27, 3.36), and among those who perceive their health as fair or poor (aOR = 2.19, 95% CI = 1.42, 3.38). Heavy SSB consumption was also significantly associated with drinking less than eight cups of water per day (aOR = 2.12 with 95% CI = 1.41, 3.18, Table 4).

## Discussion

In this study of factors associated with SSB-related knowledge, attitudes and behaviors, we found differences in consumption rates before and after a health communication program specifically designed to raise awareness and encourage behavior change related to beverage choice. Although the sample for our 2017 cross-sectional study is independent of our 2015 sample [19], the populations were only statistically different in two demographic areas, suggesting comparison between years is appropriate. Additionally, weighting allowed the sample to adequately represent the population of the State of Oklahoma [22, 23]. Results indicate SSB consumption by Oklahoma adults with children in the home decreased overall by 19% following the launch of the SYF/RYD campaign. Subgroup analysis of SSB consumption before and after the launch of the SYF/RYD campaign resulted in small sample sizes, which precluded our ability to make some subgroup comparisons. Those with less than a high school education experienced a 31% decline in SSB consumption, as did those who reported drinking eight or more cups of water per day. Similarly, good or better health status, those consuming fewer than three servings of fruit per day and those less likely to afford healthy food experienced significant declines in SSB consumption following the launch of the SYF/RYD campaign.

The aim of the SYF/RYD ad campaign was to expose adults with children in the home to messages designed to encourage replacing SSBs with water. Following the launch of the campaign in 2017, 23% of Oklahomans with children in the home demonstrated confirmed exposure, meaning they accurately recalled and described one or more SYF/RYD campaign messages. The campaign reached Oklahomans somewhat equally. While women in our study demonstrated greater confirmed exposure than men, in other categories including age, education, general health, and the ability to afford healthy food, there were no statistically significant differences in confirmed exposure. Although not statistically significant, American Indian/Alaska Natives had the lowest levels of confirmed exposure to SYF/RYD at 14%. Interestingly, this priority population, with significant obesity burden among adults and children [29] also had the highest odds of SSB consumption, and this independent association was consistent across all four SSB outcomes. Although this study did not demonstrate an association between SYF/RYD confirmed exposure and SSB consumption following program implementation, knowledge about the health consequences of SSB was statistically significantly higher among those with confirmed exposure compared to those without. It is also important to note that confirmed exposure to the SYF/RYD messages in our study was lower than reported in similar studies, about half that reported by Farley and associates (54%), although they included aided recall [11]. Similarly, Barragan and associates reported 57% exposure to billboards and other media, as well as 36% recall of television advertisements in their study [15]. The relatively low levels of confirmed campaign exposure likely contributed to this study’s inability to demonstrate an association between SSB consumption and confirmed exposure.

Research suggests dietary habits learned in childhood have a direct impact on adult eating habits and adult obesity [4, 5, 7]. Our study is especially important because every respondent had one or more children living in the home with the potential to be influenced by foods and drinks their caregivers offer, and by dietary choices their caregivers make. Zahid and associates found that home availability of SSBs was positively associated with child SSB intake (OR = 1.48 *p* = 0.03) [4]. Our study demonstrated consistent relationships between SSB consumption and other dietary behaviors such as not drinking eight or more cups of water per day and eating less than three servings of fruits and vegetables each day. An intervention that encourages parents to adopt healthy eating patterns and provide healthy foods and drinks can contribute to a reduction in SSB consumption for children in the home [30]. In addition, mass media campaigns are effective in changing beverage choices for adults [12, 14, 16] and decreasing SSB sales [11, 13]. The results of our cross-sectional study in Oklahoma are mixed when compared to those reported by other states attempting to decrease SSB consumption using mass media campaigns [12–14, 16]. For example, our study found a 19% decrease in the proportion of adults drinking SSBs daily, compared to the 35% decrease found by the city of New York after their “Pouring on the Pounds” campaign. The larger decrease seen there is likely due to an excise tax and cap on SSB portion sizes which were implemented around the same time [14]. A mass media campaign targeting parts of Tennessee, Kentucky, and Virginia in 2017 reported a 3.4% decrease in SSB sales and a 4.1% decrease in soda sales along with an overall increase in self-reported SSB consumption, following the campaign [11]. The incorporation of sales data may be a more accurate measure of SSB consumption changes over time as compared to subjective self-reports by individuals.

An important and consistent finding in this study is the association between drinking water and SSB consumption across all four definitions of the SSB outcome. The odds of drinking one or more SSB daily were about twice as high among those not drinking the recommended eight cups of water per day compared to those who drink eight or more cups of water per day. Similar to the study reported by Boles and associates [12], this finding suggests messages promoting a higher level of daily water intake could decrease SSB consumption, affecting caloric intake. In a meta-analysis using literature published from 1990 through 2014, Vargas-Garcia and associates found a paucity of research about the association between water consumption and SSB consumption in adults; this study adds to that body of evidence [17]. Additionally, two studies completed with children suggest reducing SSB consumption is associated with an increase in water consumption [17, 19]. These findings support strategies like cold water bottle re-fill stations in schools and other public places.

Rates of obesity are rising disproportionately in youth and children, as compared to adults, leading public health officials to look for solutions. While changes in dietary habits are important, they often happen in stages. Interventions may not lead to immediate changes in SSB consumption, but may have longer lasting benefits. In this study we have unearthed important findings. More than half of participants responded they intend to decrease SSB for their families (51%), and substitute water instead (59%). Additionally, about two-thirds understand the link between SSB and heart disease, diabetes, and obesity (67%). These positive steps toward change compare favorably to a study by Boles and associates in which 80% of respondents intended to reduce SSB consumption, as well as to limit SSBs offered to a child [12].

### Strengths and limitations

A strength of this study includes measurement of SSB consumption before and after the launch of the program and a unique study population – adults with at least one child in the household. Further, the definition of SSB consumption in our study allowed us to explore consumption of all SSBs as well as consumption specific to soda and beverages other than soda such as sports drinks, juice with added sugar and sugar sweetened coffee and tea. The additional category of heavy SSB consumption allowed us to demonstrate consistent, and even stronger association between important covariates such as American Indian race, perceived health status and water consumption. We also recognize the potential limitations of our study. As with any survey study, the data presented here are prone to bias, particularly recall bias. Our study is also limited to SSB consumption in adults with children in the household; we did not measure consumption among children. As a cross-sectional study, outcome, campaign exposure and covariates were assessed at the same time, thus no causal assumptions can be made. We also recognize the possibility of Type I errors due to multiple comparisons. Finally, as is typical with most evaluations of health communication campaigns, this uncontrolled design cannot attribute any change in SSB consumption directly to the SYF/RYD program.

## Conclusions

Health communication and mass media campaigns are considered an evidence-based practice for improving knowledge, attitudes, and behaviors. The Shape Your Future/Rethink Your Drink campaign in Oklahoma occurred in tandem with a significant decrease in SSB consumption among adults with children in the home. Understanding factors associated with the consumption of SSBs will lead to more effective strategies and the identification of priority populations. Continued targeted intervention to make adults with children in the home aware of the association between SSBs and obesity is an important step toward continuing the downward prevalence of SSB consumption and to reverse the trends in childhood and adult obesity.

## Data Availability

The datasets generated and/or analyzed during the current study are not publicly available due to confidentiality of respondent’s information, but are available from the corresponding author on reasonable request.

## Abbreviations

(SYF/RYD): Shape Your Future – Rethink Your Drink
SSB: Sugar-sweetened beverage
AOR: Adjusted odds ratio
OR: Odds ratio
CI: Confidence interval
BRFSS: Behavioral Risk Factor Surveillance System
TSET: Oklahoma Tobacco Settlement Endowment Trust
SS: Sugar sweetened
AAPOR: American Association for Public Opinion Research
RR1: Response rate
*p*-value: probability value
